# Bone Marrow Stromal Cell Antigen 2: Is a Potential Neuroinflammation Biomarker of SOD1^G93A^ Mouse Model of Amyotrophic Lateral Sclerosis in Pre-symptomatic Stage

**DOI:** 10.3389/fnins.2021.788730

**Published:** 2022-02-07

**Authors:** Xiaojiao Xu, Jingjing Zhang, Song Li, Murad Al-Nusaif, Qinming Zhou, Sheng Chen, Weidong Le

**Affiliations:** ^1^Institute of Neurology, Sichuan Academy of Medical Sciences and Sichuan Provincial People’s Hospital, University of Electronic Science and Technology of China, Chengdu, China; ^2^Center for Clinical Research on Neurological Diseases, The First Affiliated Hospital, Dalian Medical University, Dalian, China; ^3^Department of Neurology, Ruijin Hospital, Shanghai Jiao Tong University School of Medicine, Shanghai, China

**Keywords:** amyotrophic lateral sclerosis, SOD1^G93A^ mutation, RNA-seq, neuroinflammation, BST2

## Abstract

Neuroinflammation has long been thought to be associated with amyotrophic lateral sclerosis (ALS) development and progression. However, the exact molecular mechanisms of neuroinflammation underlying ALS remain largely unknown. In the present study, we attempted to elucidate the genetic basis of neuroinflammation in ALS by comparing the transcriptomic profile of the anterior horns of the lumbar spinal cord (AHLSC) between SOD1^G93A^ mice and their wild-type (WT) littermates. Our results revealed that immune-related genes were selectively up-regulated in the AHLSC of pre-symptomatic ALS mice (40 days of age) compared to age-matched WT control mice. Notably, the differential expression level of these immune-related genes became more significant at the symptomatic stage of disease (90 days of age) in the ALS mice. Subsequently, eight genes involved in innate immune response in the AHLSC of ALS mice were further validated by qRT-PCR analysis. Of these genes, bone marrow stromal cell antigen 2 (BST2) was found for the first time to be significantly higher in the AHLSC of pre-symptomatic ALS mice when compared with WT mice. The increasing trend of BST2 expression became more obvious in the symptomatic stage. Immunofluorescent staining further confirmed that BST2 is mainly expressed on microglia in the AHLSC of ALS mice. These findings support the view that immune-related neuroinflammation is involved in the early pathogenesis of ALS, and BST2 may serve as a potential target for ameliorating microglia-mediated neuroinflammation pathologies in ALS.

## Background

Amyotrophic lateral sclerosis (ALS) is a fatal neurodegenerative disease characterized by the progressive loss of upper and lower motor neurons (MNs) (Brown and Al-Chalabi, 2017). The average age of disease onset of ALS is 40–70 years, and the clinical manifestations may include limb weakness, muscle atrophy, dysphagia, dysarthria, etc. Respiratory failure caused by respiratory muscle weakness is the most common cause of death in the late stages of ALS. The majority (more than 90%) of ALS cases are sporadic, whereas a minor fraction (about 5–10%) is familial. Mutations in Cu/Zn superoxide dismutase 1 (SOD1), the first identified gene in ALS, characterize more than 20% of familial and 1–4% of sporadic ALS cases (Liu et al., 2018). Due to the obscure etiology and complex mechanism of ALS, effective prevention measures and treatments are still absent in clinical practice. In this way, the exploration of early diagnostic biomarkers and specific therapeutic targets are urgently needed.

Neuroinflammation is one of the most critical pathological features of ALS, characterized by glial cells activation, peripheral immune cells infiltration, and elevated secretion of inflammatory mediators. The abnormal neuroinflammatory response has been identified in regions of MNs degeneration in both ALS patients and animal models (Puentes et al., 2016). Previous studies have documented that the overexpression of the SOD1 gene with the ALS responsible mutation in MNs is insufficient to induce the pathogenesis of ALS. In contrast, overexpression of mutant SOD1 in microglia can induce significant MNs injury (Pramatarova et al., 2001; Puentes et al., 2016). Several newly discovered ALS responsible mutant genes such as *C9Orf72*, *TBK1*, *OPTN*, *VCP*, *SQSTM1*, etc., could regulate microglial activation, increase neuroinflammatory response, and MNs damage (Haukedal and Freude, 2019). Moreover, astrocytes derived from the spinal cord of ALS patients were cytotoxic to MNs in culture (Meyer et al., 2014). Worth noting, neuroinflammation can exhibit either protective or toxic effects, depending on the progression stages of the disease. Activation of microglia is a common feature of neurodegenerative diseases. The two types of microglial activation states are M1 and M2. M1 microglia could be induced by lipopolysaccharide, IFN-γ, etc., and cause neurotoxicity. In addition, IL-4 may stimulate M2 to produce anti-inflammatory substances (Lan et al., 2017). Previous studies showed that microglia were mainly polarized into the M2 phenotype to release anti-inflammatory factors at the early stage (Liao et al., 2012; Shang et al., 2020). However, during the symptomatic stage, more microglia were polarized into the M1 phenotype to increase the release of a neurotoxic substance (Liao et al., 2012; Shang et al., 2020). Restoring the balance between protective and cytotoxic effects of neuroinflammation is a direction worth exploring for ALS treatment.

In this study, we collected the anterior horns of the lumbar spinal cord (AHLSC) from the SOD1^G93A^ transgenic mouse model of ALS in both pre-symptomatic (40 days of age) and symptomatic stage (90 days), and their age-matched wild-type (WT) littermates. All samples were subjected to transcriptome analysis, and the results were further verified by qRT-PCR and/or immunofluorescent staining. We found that inflammatory and immune response genes in the AHLSC were up-regulated in SOD1^G93A^ mice. Moreover, bone marrow stromal cell antigen 2 (BST2) expression in microglia cells was significantly increased during the pre-symptomatic stage and further exacerbated after the disease onset, implying that BST2 is a key mediator and potential candidate target for ALS diagnosis and therapy.

## Results

### SOD1^G93A^ Activates Neuroinflammation in the Anterior Horns of the Lumbar Spinal Cord of Amyotrophic Lateral Sclerosis Mice in the Pre-symptomatic Stage

**FIGURE 1:**
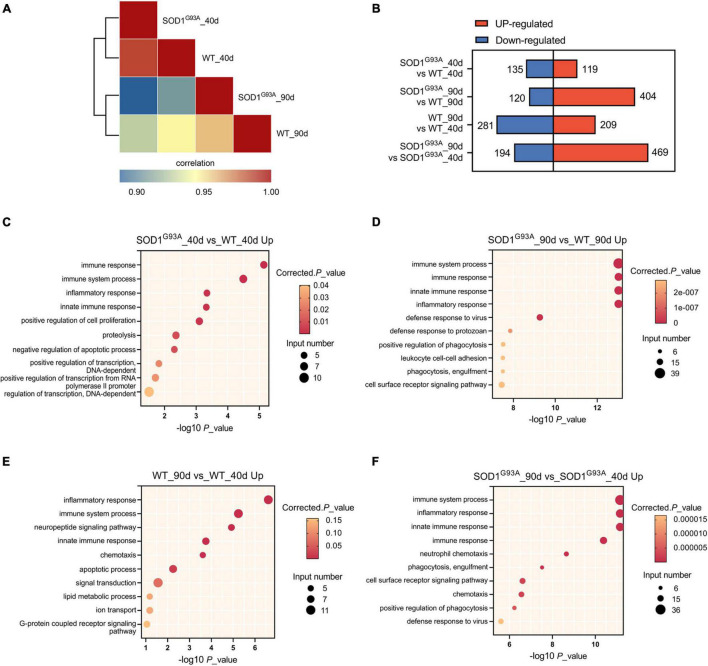
Gene expression regulation by SOD1^G93A^ mutant in mouse AHLSC. **(A)** Heat map shows the hierarchically clustered Pearson’s correlation matrix through comparing the transcript expression values. **(B)** Bar plot illustrates the up- and down-regulated gene numbers between the SOD1^G93A^ ALS and WT mice, and between symptomatic and pre-symptomatic age. **(C,D)** The top 10 representatives GO biological process terms of the SOD1^G93A^ up-regulated DEGs. **(E,F)** The top 10 representative representatives GO biological process terms of the age up-regulated DEGs.

To determine how the human mutant SOD^G93A^ affects the transcriptome of ALS mice in the pre-symptomatic and symptomatic stages, we performed transcriptome analysis of AHLSC at 40 and 90 days of age, respectively (Supplementary Table 2). The transcriptome differences between SOD^G93A^ and WT mice were presented at the pre-symptomatic stage (40 days of age) and became more pronounced at the symptomatic stage (90 days of age) (Figure 1A). EdgeR in the setting of a GLM was used to identify differential expression genes (DEGs). 254 and 524 genes were found differentially expressed between SOD1^G93A^ mice and WT mice in the AHLSC of 40 and 90 days, respectively (Figure 1B).

To further understand the function of these abnormal expression genes in ALS, Gene Ontology (GO) enrichment analysis was performed. At the pre-symptomatic stage, the top four GO biological process terms enriched by the 119 up-regulated genes were immune response, immune system process, innate immune response, and inflammatory response (Figure 1C). Meanwhile, the top 1 process enriched by the 135 down-regulated genes in AHLSC of 40 days SOD1^G93A^ mice was also immune-related (Supplementary Figure 1A). A growing body of evidence demonstrated genetics of neurodegenerative illnesses was linked to altered immune signaling. Specifically, significant enrichment of immune signaling and expression within microglia and macrophages in Alzheimer’s disease risk loci were repeatedly reported (Lambert et al., 2009, 2013; Hollingworth et al., 2011; Hammond et al., 2019; Kunkle et al., 2019). Considering that inflammation is a central characteristic of an effective immune response (Brady et al., 2018; McCauley and Baloh, 2019), these results might indicate that SOD1^G93A^ can selectively influence the neuroinflammation gene expressions of ALS mice in the early stage.

According to the results of GO enrichment, immune response, immune system process, innate immune response, and inflammatory response were all significantly enriched by up-regulated genes and became much more prominent in the symptomatic stage (Figure 1D) in the symptomatic stage. Furthermore, compared 90 days-aged SOD1*^G93A^* mice to 40 days-aged SOD1^G93A^ mice, these processes were also significantly enriched (Figure 1F), indicating that aberrant immunological signaling plays a vital role in the pathophysiologic alterations associated with ALS. Other immune-related biological processes such as defense response to the virus and protozoan, as well as phagocytosis, a function of macrophage and microglia, were also enriched by the 404 up-regulated genes (Figure 1D). Whereas, no immune-related process was enriched by the 120 down-regulated genes in SOD1^G93A^ mice at 90 days of age (Supplementary Figure 1B), suggesting that none of the down-regulated genes present at the onset stage are involved in the immune response process. These findings further indicate that immune-related DEGs between WT and SOD1^G93A^ mice at the symptomatic stage are systematically up-regulated. Simultaneously, inflammatory response, immune system process, and innate immune response were also enriched by the differential metabolites between 40 and 90 days’ WT mice (Figure 1E), which demonstrated the age-dependent feature of immune signaling.

### Several Inflammatory Genes Expression Significantly Increased in the Anterior Horns of the Lumbar Spinal Cord of Pre-symptomatic SOD1^G93A^ Mice

**FIGURE 2:**
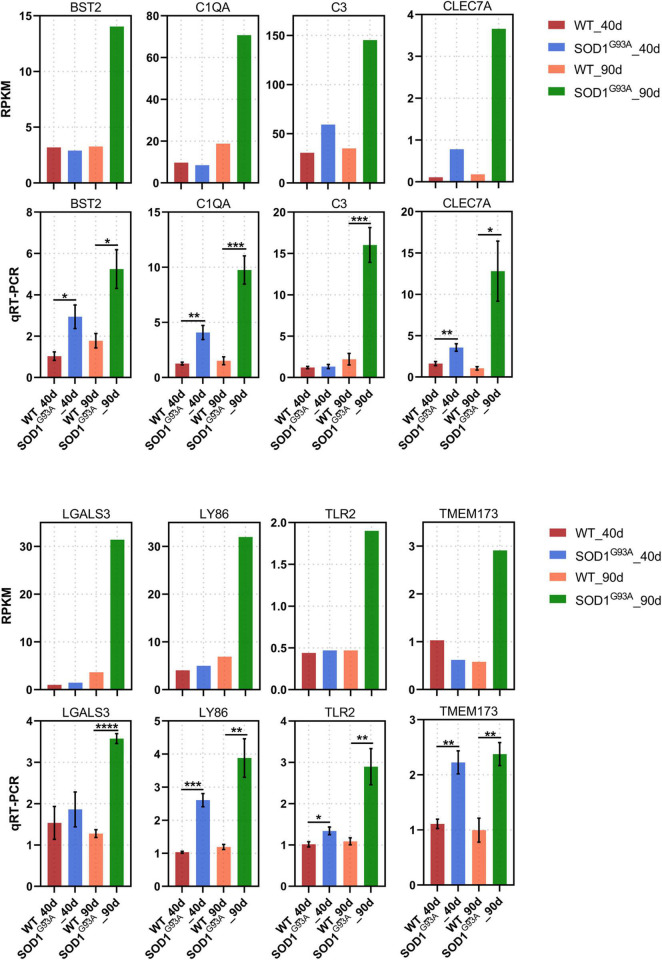
Neuroinflammation gene expression increased in AHLSC of SOD^G93A^ mouse. Relative expression levels of 8 inflammation genes were measured by RNA-seq (RPKM) (up) and qRT-PCR (down). GAPDH was used as the reference gene for qPCR, and WT samples were used as the control groups, *p < 0.05 versus age-matched WT mice, ** *p* < 0.01 versus age-matched WT mice, ***p < 0.001 versus age-matched WT mice, ****p < 0.0001 versus age-matched WT mice, *n* = 4 in each group.

Of the 404 up-regulated genes in the AHLSC of SOD1^G93A^ mice at 90 days of age, 65 genes were enriched in the biological processes of the immune response, immune system process, innate immune response, and inflammatory immune response (Supplementary Table 3). And 26 genes were found to be involved in innate immune response (Supplementary Table 4). To confirm the reliability of the RNA-seq and further investigate the trend of specific genes involved in innate immune response, eight genes, including C-Type Lectin Domain Containing 7A (CLEC7A), toll-like receptor 2 (TLR2), complement C1q A chain (C1QA), complement C3 (C3), galectin 3 (LGALS3), transmembrane protein 173 (TMEM173), lymphocyte antigen 86 (LY86), and BST2 were selected from 26 DEGs which were enriched in innate immune response due to their reported correlation with two or more neurologic disorders (Supplementary Figure 2 and Supplementary Table 4). Simultaneously, the RPKM of these selected genes is required to be greater than 0.1 in both the WT and SOD1^G93A^ mice (Supplementary Table 4). Consistent with the results of RNA-seq, mRNA levels of all these eight genes were significantly elevated in the AHLSC of SOD1^G93A^ mice after disease onset (Figure 2). Moreover, mRNA levels of BST2, C1QA, CLEC7A, TLR2, LY86, and TMEM173 were significantly elevated since the pre-symptomatic stage in the AHLSC of SOD1^G93A^ mice, and became highly significant after disease onset. Since most MNs loss in the AHLSC of SOD1^G93A^ occurs at the onset of symptoms, these findings may imply that neuroinflammation plays an important role in the early stages of ALS progression (Chiu et al., 1995).

### The Level of Bone Marrow Stromal Cell Antigen 2, Galectin 3, and Toll-Like Receptor 2 Altered in Anterior Horns of the Lumbar Spinal Cord, and Bone Marrow Stromal Cell Antigen 2 Was Significantly Elevated at the Pre-symptomatic Stage in Microglia

Neuroinflammation mediated by microglia and astrocytes is the primarily pathophysiological mechanism implicated in ALS. Thus, we tried to investigate the relationship between these innate immune-related genes and glial cells activation. Among these eight genes, we chose BST2, LGALS3, and TLR2 for further study. BST2, LGALS3, and TLR2 have been reported to involve in the innate immune responses in CNS. However, their relationship with glial activation in ALS *in vivo* remains unknown. Additionally, accumulating data suggest that LGALS3 might become a biomarker candidate due to its consistent alteration in CSF and spinal ALS patients (Zhou et al., 2010). TLR2 could mediate microglia activation when induced by mutant SOD1 *in vitro* (Kinsella et al., 2016). And BST2 was demonstrated in MS that only activated microglia express it (Manouchehri et al., 2021).

**FIGURE 3:**
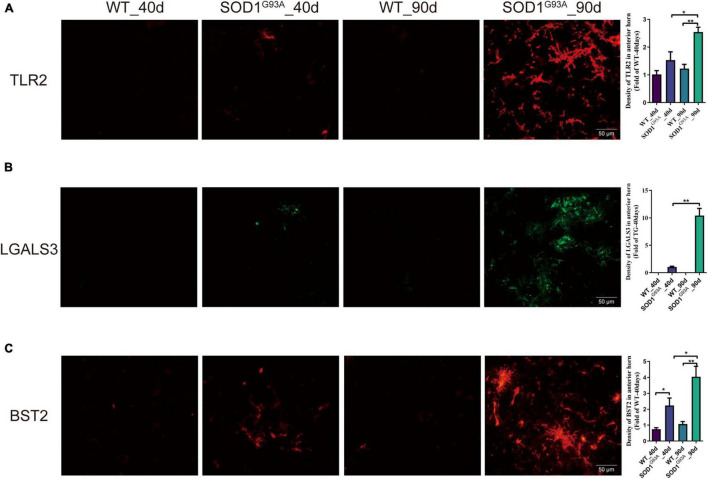
The expression of TLR2, BST2, and LGALS3 protein in AHLSC of SOD1^G93A^ mouse. Immunofluorescence staining and intensity analysis of TLR2 **(A)**, LGALS3 **(B)**, and BST2 **(C)** in the AHLSC of 40- and 90-day ALS mice. The right panel shows the quantitative results, *p < 0.05, ** *p* < 0.01, Scale Bar = 50 μm. The values are expressed as mean ± SD, *p < 0.05 versus age-matched WT mice, **p < 0.01 versus age-matched WT mice, *n* = 4 in each group.

Immunofluorescent staining revealed a significant increase in the immunofluorescent intensity of TLR2, BST2, and LGALS3 in the AHLSC of SOD1^G93A^ mice at 90 days of age as compared to WT mice (Figure 3), consistent with the RNA-seq results. More importantly, immunofluorescent staining also showed that the expression of BST2 and LGALS3 had significantly increased on days 40, the early stage of SOD1^G93A^ mice disease progression (Figures 3B,C), while the elevated expression of TLR2 was observed on days 90, in the symptomatic stage (Figure 3A).

**FIGURE 4:**
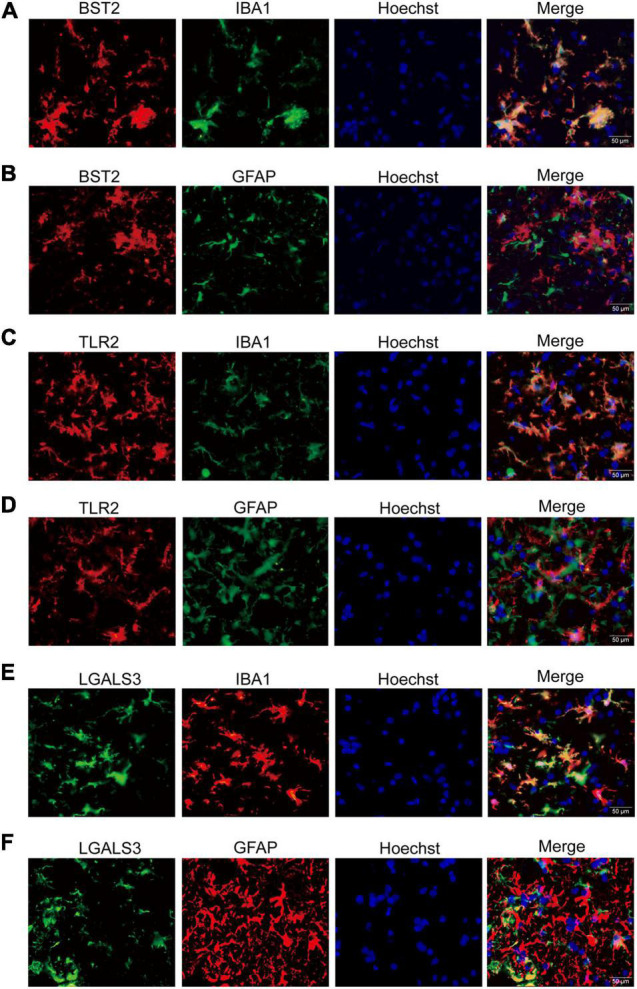
The immunofluorescent staining of BST2, TLR2, and LGALS3 on microglia or astrocytes. **(A,B)** Double immunofluorescent staining of BST2 in the AHLSC of 90-day ALS mice. The Iba1-labeled microglia **(A)** and GFAP-labeled astrocyte **(B)** in 90-day ALS mice. **(C,D)** The results for TLR2. **(E,F)** The results for LGALS3; Scale bar = 50 μm.

Interestingly, the co-immunofluorescent staining showed that TLR2 and BST2 were mainly expressed in microglia in the AHLSC region of mice (Figures 4A–D), while LGALS3 was expressed in both microglia and astrocytes (Figures 4E,F). All results indicate that the increase in inflammation and immune-related genes might induce the abnormal proliferation and activation of microglia and astrocytes. On the other hand, aberrant glial cells activation might aggravate neuroimmune disturbances by releasing detrimental inflammatory mediators. Based on these results, it is reasonable to assume that BST2, TLR2, and LGALS3 are implicated in the abnormal activation of microglia or astrocytes and eventually lead to the neurotoxic effect causing impairment MNs during ALS disease progression.

Despite the contributions of astrocytes, oligodendrocytes, and other cells, the previous study indicates that microglia play a critical role in MN degeneration and disease pathogenesis (Lall and Baloh, 2017). It was confirmed that abnormal activation of microglia was evident before the onset of symptoms and exaggerated with the progression of ALS (Hall et al., 1998; Alexianu et al., 2001). The correlation between BST2 and ALS has never been elucidated in previous studies. Our study found that the expression of BST2 elevated in AHLSC at 40 days of age in SOD1^G93A^ mice and further increased as the disease progressed.

Moreover, BST2 was specifically localized in microglia in AHLSC, and its alteration of expression level corresponded to the abnormal activation of microglia. Thus, we speculated that BST2 might play an essential role in developing neuroinflammatory pathologies of ALS. And it might become a promising target to redirect microglia-mediated neuroinflammation for ALS therapy.

## Discussion

Previous studies have demonstrated that abnormal neuroinflammation plays a critical role in the pathogenesis and progression of ALS. Moreover, while some anti-inflammatory medications showed promising results in preclinical studies, none of them improved the outcomes of ALS patients in clinical trials (Rizzo et al., 2014). The heterogeneity of neuroinflammatory responses might account for the failure of certain anti-inflammatory compounds in clinical use. Several complicated signaling pathways have been proposed to be involved in neuroinflammation regulation. And whether neuroinflammation exerted a protective or detrimental effect mainly depended on the disease stage and the activated types of glial cells. Understanding the mechanism to regulate the switch from a neurotoxic phenotype to a neurosupportive one is essential for developing efficacious therapies. In other words, elucidation of the transforming mechanism of neuroinflammatory response could guide future treatments in ALS (Cipollina et al., 2020).

This study used RNA-Seq to evaluate transcriptomic changes in AHLSC of SOD1^G93A^ mice compared with age-matched WT mice at the pre-symptomatic (40 days) and symptomatic (90 days) stages of the disease. We found that human SOD1^G93A^ mutation can mainly influence the immune and neuroinflammation processes, including immune response, immune system process, inflammatory response, and innate immune response in the AHLSC of pre-symptomatic and symptomatic ALS mice. Although there have been some transcriptome studies about SOD1^G93A^ mice previously (Chen et al., 2010; Bandyopadhyay et al., 2013; Nardo et al., 2013; Holtman et al., 2015), most studies only focus specifically on MNs or glial cells at the symptomatic stage, and few of them investigated alterations in pre-symptomatic SOD1^G93A^ mice. As AHLSC is the primary lesion site of symptomatic ALS mice, glial cells, and MNs present simultaneously in this region, cellular interactions should not be overlooked during ALS progression (Brites and Vaz, 2014). RNA-seq results in our study revealed that immune-related processes played a critical role in neuroinflammation in the AHLSC of ALS mice before and during disease onset. More importantly, one recent spatial transcriptomic analysis of the ALS mice spinal cords revealed that microglia dysfunction is evident at the age of 30 days, which would then gradually lead to astrocyte dysfunction and neuronal death, further suggesting the importance of neuroinflammation in ALS pathophysiological manifestations (Maniatis et al., 2019).

It has been reported that several inflammatory genes are involved in the pathogenesis of ALS. In our study, 65 of 524 DEGs of symptomatic ALS mice were enriched in immune and neuroinflammation-related biological processes. And there were 26 genes involved in innate immune response. In order to explore the correlation between innate immune response, we chose 8 of them: C1QA, C3, TLR2, LGALS3, LY86, CLEC7A, TMEM173, and BST2 for qRT-PCR analysis. As expected, all of them had a prominent elevation at the symptomatic stage of SOD1^G93A^ mice. The expression levels of BST2, CLEC7A, TLR2, C1QA, LY86, and TMEM173 were significantly increased after 40 days of age.

As the classic complement pathway components, C3 and C1QA could activate microglia to drive synapse elimination and cell death through microglial complement receptor CR3 (Hong et al., 2016). Although accumulating data suggests that C3 and C1QA regulation might be the target of mitigating microglia toxicity and neurodegeneration, no therapeutic effect was observed in C1Q, or C3 gene deleted SOD1^*G*37*R*^ mice model (Lobsiger et al., 2013; Zhang et al., 2020), which demonstrated that activating a complement pathway containing C1Q and C3 might not be the major part of ALS pathogenesis in mice. Increased expression of TLR2 was found in the spinal cord of ALS patients (Casula et al., 2011). Moreover, *in vitro* study found that mutant SOD1 could activate microglia through TLR2 receptors to promote the release of pro-inflammatory factors, and blocking TLR2 receptor could attenuate microglial activation, which indicates that regulation of TLR2 might benefit in reducing inflammatory responses (Zhao et al., 2010). CLEC7A is a disease-associated microglia marker reported in AD and ALS. And it is known to exhibit anti-inflammation and neuroprotective effects in the CNS (Keren-Shaul et al., 2017; Deerhake and Shinohara, 2021; Hunter et al., 2021). LGALS3 is a multifunctional immunomodulatory lectin that is expressed by a variety of cells throughout normal development as well as during inflammatory and autoimmune disorders. Elevated LGALS3 level has been reported in SOD1^G93A^ mice and sporadic ALS patients’ spinal cord and plasma (Lerman et al., 2012; Yan et al., 2016). Previous studies documented that knocking out LGALS3 would cause excessive activation of microglia and aggravate the progression in the ALS mice. Thus researchers speculated that elevated LGALS3 might exert a protective effect on MNs (Lerman et al., 2012).

The changes of LY86, TMEM173, and BST2 have not been reported in ALS. However, there are rare studies to elucidate the functions of LY86 (also known as MD1) and TMEM173 in CNS diseases. And no study has documented the correlation between TMEM173 or LY86 and glial activation mediated by the innate immune response. In the immune response, LY86 (MD1) requires a tight combination with RP105 (a TLR-like, cell surface molecule) to regulate TLR4 signaling and induce an innate immune response (Yoon et al., 2011; Watanabe et al., 2012). Thus, studies toward the relationship between LY86 and neuroinflammation need to distinguish the function of the LY86 and RP105-LY86 complex. TMEM173, also known as Sting, could activate down-streaming NF-κB and MAPKs signaling pathways (Wang et al., 2019; Balka et al., 2020), which indicates that TMEM173 might induce glial activation mainly through NF-κB. And the interaction of NF-κB and microglia activation has been extensively studied in previous studies (Frakes et al., 2014; Parisi et al., 2016; Dokalis and Prinz, 2018). Moreover, our study aimed to explore the relationship between innate immune-related molecules and neuroinflammation mediated by glial cells. Considering the research status and pathological features of chosen genes, thus, we focused on the alteration of BST2 in the pathological changes of the SOD1^G93A^ mice model.

In our present study, we found for the first time that the expression of BST2 significantly increased in the AHLSC of ALS mice starting at the pre-symptomatic stage, and further increased as the disease progressed. The immunofluorescent staining showed that BST2 was mainly expressed in microglia in the AHLSC region of mice. Based on these data, we speculated that BST2 had a strong correlation with the activation of microglia. BST2, also known as Tetherin or CD317, is a type II transmembrane protein located on the cell membrane. It can work as a negative regulator of type I IFN signaling and down-regulate the expression of IFN-α and TNF-α (Jin et al., 2017). The expression of BST2 could be induced by signal transducer and activator of transcription (STAT) after the stimulation from type I and type II IFNs (Blasius et al., 2006). Simultaneously, pSTAT3 was reported to express in glial cells in the spinal cord of ALS patients and animal models of ALS. The specific processes of BST2 activation and interaction with STAT3 remain unclear, and the role and an explicit function of BST2 still need further investigation (Ohgomori et al., 2017).

Microglia-like cells exhibited concurrent expression of BST2 in human CSF fluid from patients with neuroinflammation, whereas BST2 was expressed in only activated microglia in mice (Manouchehri et al., 2021). Oxidative injury is a pathologic feature in neurodegenerative diseases mediated by the innate immune response. To figure out the transcriptomic landscape of innate immune cells implicated in oxidative stress in CNS, a single-cell RNA-seq transcriptional profiling of ROS^+^ CNS innate immune cells was carried out. According to the reference, it was found that BST2 was highly expressed in 2 microglia clusters of EAE, this indicates that BST2 might promote oxidative stress in microglia and lead to abnormal activation (Mendiola et al., 2020). In the experimental EAE model, anti-BST2 treatment before clinical EAE could significantly antagonize the disease-propagating effect and improve recovery by reducing mediators of persistent clinical EAE in the CNS. Moreover, blocking down the expression of BST2 can improve the symptom of the EAE mice (Manouchehri et al., 2021). All these findings may demonstrate the close relationship between BST2 and inflammatory response. Considering the crucial role of neuroinflammation in ALS pathogenesis, the correlation between BST2 and ALS progression is worth further investigation. Taken together, we speculated that elevation of BST2 expression in SOD1^G93A^ mice since the pre-symptomatic stage correlated with abnormal microglial activation. BST2 may be a candidate biomarker for ALS neuroinflammation, and anti-BST2 therapy may be a promising strategy in delaying ALS onset and prolonging survival.

## Conclusion

In our study, four biological processes, including immune response, immune system process, inflammatory response, and innate immune response, were up-regulated in the AHLSC of ALS mice both at the pre-symptomatic and symptomatic stages. Eight of the genes involved in innate immune response were validated by qRT-PCR. And the qRT-PCR results confirmed that the expression of BST2, CLEC7A, TLR2, C1QA, LY86, and TMEM173 significantly elevated since day 40. Moreover, the co-expression of BST2 with microglia and the significantly elevated expression level in the AHLSC of ALS mice since the pre-symptomatic stage were also found by immunofluorescent staining. Thus, we suggest that BST2 might have the great potential to be used as the target to ameliorate neuroinflammation pathologies in ALS, specifically caused by the activation of microglia. Furthermore, considering the of anti-BST2 treatment on EAE, it might provide ideas for developing new therapeutic strategies for ALS in the future.

## Materials and Methods

### Animals and Tissue Extraction

The heterozygous SOD1^G93A^ transgenic mice expressing about 20 copies of mutant human SOD1 with a Gly93Ala substitution were obtained from the Jackson Laboratory (B6SJLT-Tg-SOD1-G93A-1Gur, 002726). Breeding was generated by crossing male SOD1^G93A^ hemizygous with WT females of the same B6SJL genetic background and housed in a temperature-controlled room (22°C) with a light-dark 12:12 cycle (lights on 07:00–19:00 h) at the Dalian Medical University. PCR identified the genotype of the TG mice as our previous report (Zhang et al., 2018). ALS mice usually began to show initial symptoms of MNs loss at around 90 days and hind limbs paralysis at around 120 days. We selected pre-symptomatic or symptomatic TG mice and age matching WT mice as the control group. At the two time-points of 40 and 90 days of age, the experimental mice were anesthetized with 10% chloral hydrate; their hearts were perfused with 0.1 M ice-cold phosphate buffer saline (PBS). Under a dissecting microscope, the lumbar spinal cord was extracted, and the surrounding tissues of AHLSC were dissected, according to the previously reported protocol (Zhang et al., 2018). Tissues were collected and stored at −80°C for subsequent molecular biological investigation. All experiments were carried out in accordance with the guidelines of the Institutional Animal Care and Use Committee. All procedures for animal experiments were approved by Dalian Medical University’s Ethics Committee.

### RNA Library Construction and Sequencing

Total RNA was extracted from AHLSC tissues with TRIzol Reagent (Invitrogen, Carlsbad, CA, United States). RQ1 DNA enzyme (Promega, United States) was used to remove DNA contamination. The RNA quality, concentration, and integrity were determined by measuring the absorbance at 260 nm/280 nm, and 1.5% agarose gel electrophoresis, 3 μg of total RNA were taken from each group to prepare the RNA-seq library. The polyadenylated mRNAs were purified and concentrated by oligonucleotide (dT)-coupled magnetic beads, then mRNAs were used to prepare the directed RNA-seq library. Purified mRNAs were iron fragmented at 95°C followed by end repair and 5′ adaptor ligation. Then reverse transcription was performed with RT primer harboring 3′ adaptor sequence and randomized hexamer. The cDNA was purified and amplified by PCR. The corresponding 200–500 bps PCR products were purified, quantified, and stored at −80°C for subsequent sequencing. For high-throughput sequencing, the libraries were prepared following the manufacturer’s instructions and applied to the Next 500 system for 150 nt pair-end sequencing by ABlife, Inc. (Wuhan, China).

### RNA-seq Data Processing and Alignment

After discarding the raw reads containing N bases, Cutadapt (Version 1.8.1) and FASTX-Toolkit (Version 0.0.13) were used to remove adaptors, low-quality bases, and short reads smaller than 16 nt from the original sequencing reads. Tophat2 was used to compare the clean reads with the GRCh38 genome (Trapnell et al., 2012). Four mismatches are allowed. The uniquely mapped reads are used to count the number of reads for each gene and calculate the RPKM value (fragments per kilobase of transcript per million fragments mapped) (Trapnell et al., 2010).

### Differentially Expressed Gene and Gene Ontology Enrichment Analysis

The R Bioconductor package EdgeR was utilized to screen out the differentially expressed genes (DEGs) (Robinson et al., 2010). A false discovery rate <0.01 and fold change ≥2 or ≤0.5 were set as the cut-off criteria for identifying DEGs. GO enrichment analysis was performed with DAVID online platform to identify significant canonical pathways in which DEGs were enriched.^1^ The enrichment of each pathway was defined by using the Hypergeometric test and Benjamini-Hochberg FDR controlling procedure.

### Quantitative Real-Time PCR

Total RNA was extracted using TRIzol reagent (Invitrogen, Carlsbad, CA, United States), and reverse transcription was performed according to the manufacturer’s instructions (638315, Clontech Laboratories, Inc., A Takara Bio Company, United States). Quantitative real-time PCR (qRT-PCR) was performed to determine the expression level of immune genes using a proper qRT-PCR primer from the cDNA Synthesis Kit as the reverse primer (A full list of qRT-PC primers information is given in Supplementary Table 1).

### Immunofluorescent Staining

TG and age-matched WT mice were sacrificed by perfusion through the heart with ice-cold 0.1 M PBS and 4% paraformaldehyde (PFA) at the age of 40 and 90 days; the enlarged lumbar regions (L4–L6) of the spinal cords were immediately removed and immersed in the same fixative overnight at 4°C. Then the spinal cords were gradiently placed in 15 and 30% sucrose solution to dehydrate for 24 h at 4°C for dehydration, frozen in OCT compound, and sectioned at 10-μm thickness in freezing microtome at −25°C. Immunofluorescent staining was performed according to standard protocols; briefly, frozen sections were baked for 30 min at 55°C and in 5% bovine serum albumin (BSA), 0.3% Triton in PBS for 1 h, and then incubated overnight at 4°C with following primary antibodies: anti-TLR2, anti-BST2, anti-LGALS3, anti-Iba1, and anti-GFAP (Antibody information is given in Table 1). After removing the primary antibodies, the sections were rinsed in PBS before being incubated for 2 h at room temperature with secondary goat anti-rabbit IgG conjugated Alexa 594 or 488, goat anti-mouse IgG conjugated Alexa 594 or 488, donkey anti-rabbit IgG conjugated Alexa 555, IFKine Green donkey anti-goat IgG, and Cy3 goat anti-rat IgG. Hoechst solution was used to stain the nuclei. A fluorescent microscope (Olympus) was used to visualize and photograph the images. The Image J software^2^ was used to calculate the integrated density of positive staining on eight slices per mouse (*n* = 4 in each group).

**TABLE 1:** Antibody information.

Antibody	Manufacturer	Catalog	Concentration for IF
TLR2	Abcam	ab11864	1;150
BST2	BioLegend	127002	1:100
LGALS3	R&D Systems	AF1197-SP	1:100
Iba1	WAKO	019-19741	1:1000
GFAP	DAKO	Z0334	1:2000
SMI-32	Abcam	ab8135	1:1000
Goat anti-rabbit IgG conjugated Alexa 594	CST	8889S	1:2000
Goat anti-rabbit IgG conjugated Alexa 488	CST	4412s	1:2000
Goat anti-mouse IgG conjugated Alexa 594	CST	8890s	1:2000
Goat anti-mouse IgG conjugated Alexa 488	CST	4408S	1:2000
Donkey anti-rabbit IgG conjugated Alexa 555	Beyotime	A0453	1:2000
IFKine™ Green donkey anti-goat IgG	Abbkine	A24231	1:500
Cy3 goat anti-rat IgG	Beyotime	A0507	1:2000

### Statistical Analysis

All the statistics were expressed as mean ± standard error (SE) and processed using R language or statistical program for social sciences (SPSS) 22.0. One-way ANOVA was used to determine whether significant differences existed between groups. All experiments were performed at least three times independently, and *p* < 0.05 was considered statistically significant.

## Data Availability Statement

The original contributions presented in the study are included in the article/Supplementary Material, further inquiries can be directed to the corresponding author/s.

## Ethics Statement

The animal study was reviewed and approved by the Ethical Committee of Dalian Medical University.

## Author Contributions

WL designed the study. WL, XX, and JZ draft the manuscript. XX, SL, and MA-N edited and finalized the manuscript. JZ, XX, and SC performed the data analysis. JZ and QZ performed the experiments. All authors contributed to the article and approved the submitted version.

## Conflict of Interest

The authors declare that the research was conducted in the absence of any commercial or financial relationships that could be construed as a potential conflict of interest.

## Publisher’s Note

All claims expressed in this article are solely those of the authors and do not necessarily represent those of their affiliated organizations, or those of the publisher, the editors and the reviewers. Any product that may be evaluated in this article, or claim that may be made by its manufacturer, is not guaranteed or endorsed by the publisher.

## Footnotes

1
https://david.ncifcrf.gov/

2
https://imagej.nih.gov/ij/

## Supplementary Material

Supplementary Figure 1 Gene Ontology (GO) enrichment analysis of down-regulated genes by SOD1^G93A^ mutant in mouse AHLSC. **(A,B)** The top 10 GO biological process terms enriched by the down-regulated DEGs: SOD1 versus WT at 40 **(A)** and 90 **(B)** days.

Supplementary Figure 2 Volcano plot.

## Abbreviations

ALS: amyotrophic lateral sclerosis
AHLSC: anterior horns of the lumbar spinal cord
BST2: bone marrow stromal cell antigen 2
MNs: motor neurons
SOD1: Cu/Zn superoxide dismutase 1
IFN: interferons
EAE: encephalomyelitis
WT: wild-type
DEGs: differential expression genes
GO: Gene Ontology
CLEC7A: C-Type Lectin Domain Containing 7A
TLR2: toll-like receptor 2
C1QA: complement C1q A chain
C3: complement C3
LGALS3: galectin 3
TMEM173: transmembrane protein 173
LY86: lymphocyte antigen 86
TBK1: TANK binding kinase 1
OPTN: optineurin
VCP: valosin containing protein
SQSTM1: sequestosome 1
STAT: signal transducer and activator of transcription.
