# Supplementary figures and images for: Bone Marrow Stromal Cell Antigen 2: Is a Potential Neuroinflammation Biomarker of SOD1G93A Mouse Model of Amyotrophic Lateral Sclerosis in Pre-symptomatic Stage
Source: Front Neurosci. 2022 Feb 7;15:788730. doi: 10.3389/fnins.2021.788730 (PMC8858987; doi:10.3389/fnins.2021.788730)

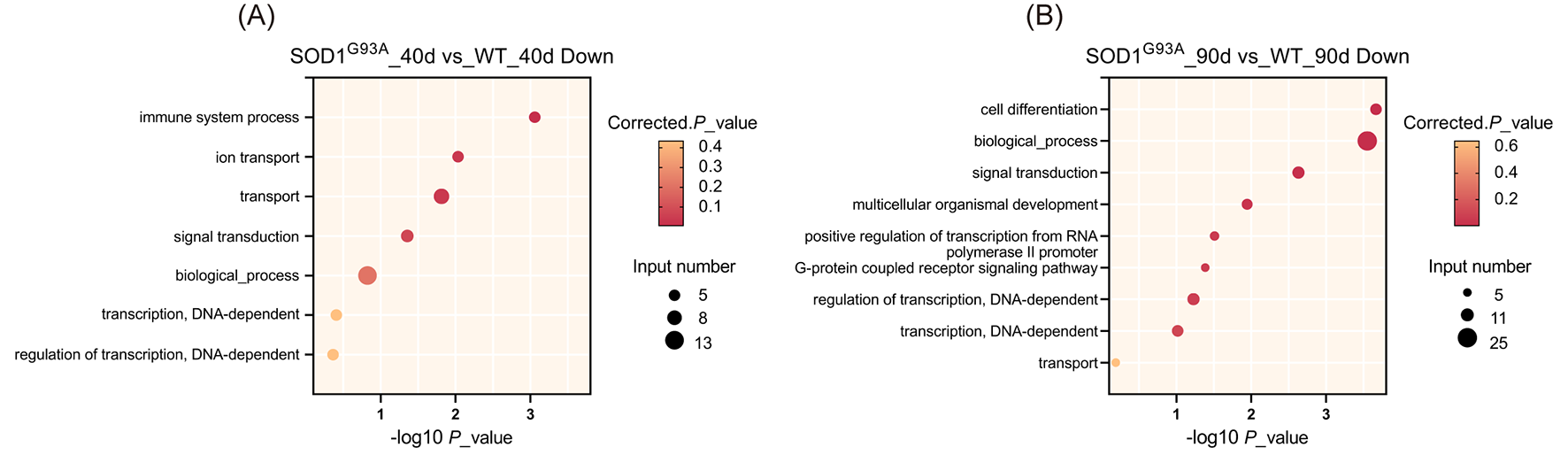

Supplement: Supplementary Figure 1 — Gene Ontology (GO) enrichment analysis of down-regulated genes by SOD1G93A mutant in mouse AHLSC. (A,B) The top 10 GO biological process terms enriched by the down-regulated DEGs: SOD1 versus WT at 40 (A) and 90 (B) days. [file Image_1.TIF]

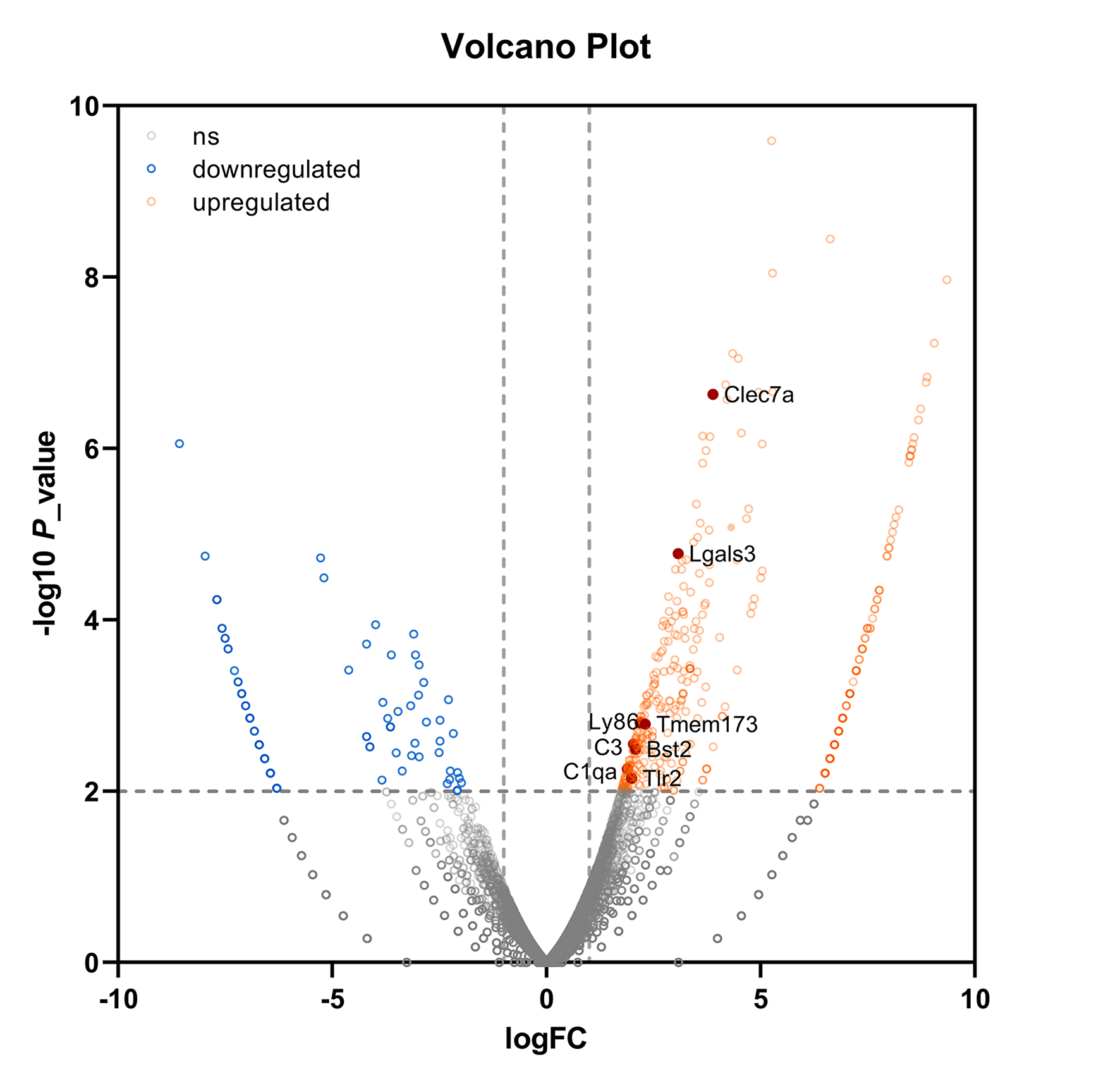

Supplement: Supplementary Figure 2 — Volcano plot. [file Image_2.TIF]
